# Replays of spatial memories suppress topological fluctuations in cognitive map

**DOI:** 10.1162/netn_a_00076

**Published:** 2019-07-01

**Authors:** Andrey Babichev, Dmitriy Morozov, Yuri Dabaghian

**Affiliations:** Department of Computational and Applied Mathematics, Rice University, Houston, TX, USA; Lawrence Berkeley National Laboratory, Berkeley, CA, USA; Department of Computational and Applied Mathematics, Rice University, Houston, TX, USA; Department of Neurology, The University of Texas McGovern Medical School, Houston, TX, USA

**Keywords:** Learning and memory, Hippocampal replays, Transient networks, Zigzag homology theory

## Abstract

The spiking activity of the hippocampal place cells plays a key role in producing and sustaining an internalized representation of the ambient space—a cognitive map. These cells do not only exhibit location-specific spiking during navigation, but also may rapidly replay the navigated routs through endogenous dynamics of the hippocampal network. Physiologically, such reactivations are viewed as manifestations of “memory replays” that help to learn new information and to consolidate previously acquired memories by reinforcing synapses in the parahippocampal networks. Below we propose a computational model of these processes that allows assessing the effect of replays on acquiring a robust topological map of the environment and demonstrate that replays may play a key role in stabilizing the hippocampal representation of space.

## Author Summary

In this manuscript, we use methods of zigzag homology theory to study the physiological role of the replays—the hippocampal networks endogenous activity that recapitulates the activity of the place cells during exploration of the environment. In particular, we demonstrate that deterioration of the hippocampal spatial memory map caused by excessive transience of synaptic connections may be mitigated by spontaneous replays. The results help to understand how transient information about local spatial connectivity may stabilize at a large scale, and shed light on the separation between faster and slower memory processing in the complementary (hippocampal and neocortical) learning systems.

## INTRODUCTION

Spatial awareness in mammals is based on an internalized representation of the environment—a cognitive map. In rodents, a key role in producing and sustaining this map is played by the hippocampal place cells, which preferentially fire action potentials as the animal navigates through specific domains of a given environment—their respective place fields. Remarkably, hippocampal place cells may also activate due to the endogenous activity of the hippocampal network during quiescent wake states (Johnson & Redish, 2007; Pastalkova, Itskov, Amarasingham, & Buzsáki, 2008) or sleep (Ji & Wilson, 2007; Louie & Wilson, 2001; Wilson & McNaughton, 1994). For example, the animal can preplay place cell sequences that represent possible future trajectories while pausing at “choice points” (Papale, Zielinski, Frank, Jadhav, & Redish, 2016), or replay sequences that recapitulate the order in which the place cells have fired during previous exploration of the environment (Foster & Wilson, 2006; Hasselmo, 2008). Moreover, spontaneous replays are also observed during active navigation, when the hippocampal network is driven both by the idiothetic (body-derived) inputs and by the network’s autonomous dynamics (Carr, Jadhav, & Frank, 2011; Dragoi & Tonegawa, 2011; Jadhav, Kemere, German, & Frank, 2012; Jadhav, Rothschild, Roumis, & Frank, 2016; Karlsson & Frank, 2009).

Neurophysiologically, place cell replays are viewed as manifestations of the animal’s “mental explorations” (Babichev & Dabaghian, 2018; Dabaghian, 2016; Hopfield, 2010; Zeithamova, Schlichting, & Preston, 2012), which help constructing the cognitive maps and consolidating memories (Ego-Stengel & Wilson, 2010; Gerrard, Kudrimoti, McNaughton, & Barnes, 2001; Girardeau, Benchenane, Wiener, Buzsáki, & Zugaro, 2009; Girardeau & Zugaro, 2011; Roux, Hu, Eichler, Stark, & Buzsáki, 2017). Although the detailed mechanisms of these phenomena remain unknown, it is believed that replays may reinforce synaptic connections that deteriorate over extended periods of inactivity (Sadowski, Jones, & Mellor, 2011, 2016; Singer, Carr, Karlsson, & Frank, 2013).

The activity-dependent changes in the hippocampal network’s synaptic architecture occur at multiple timescales (Bi & Poo, 1998; Fusi, Asaad, Miller, & Wang, 2007; Karlsson & Frank, 2008). In particular, statistical analyses of the place cells’ spiking times indicate that place cells exhibiting frequent coactivity tend to form short-lived “cell assemblies”—commonly viewed as *functionally* interconnected groups of neurons that form and disband at a timescale between tens of milliseconds (Atallah & Scanziani, 2009; Bartos, Vida, & Jonas, 2007; Buzsáki, 2010; Harris, Csicsvari, Hirase, Dragoi, & Buzsáki, 2003) to minutes or longer (Billeh, Schaub, Anastassiou, Barahona, & Koch, 2014; Goldman-Rakic, 1995; Hiratani & Fukai, 2014; Kuhl, Shah, DuBrow, & Wagner, 2010; Murre, Chessa, & Meeter, 2013; Russo & Durstewitz, 2017; Zenke & Gerstner, 2017), that is to say the functional architecture of this network is constantly changing. In our previous work (Babichev & Dabaghian, 2017a, 2017b; Babichev, Morozov, & Dabaghian, 2018) we used a computational model to demonstrate that despite the rapid rewirings, such a “transient” network can produce a stable topological map of the environment, provided that the connections’ decay rate and the parameters of spiking activity fall into the physiological range (Arai, Brandt, & Dabaghian, 2014; Basso, Arai, & Dabaghian, 2016; Dabaghian, Mémoli, Frank, & Carlsson, 2012). Below we adopt this model to study the role of the hippocampal replays in acquiring a robust cognitive map of space. Specifically, we demonstrate that reinforcing the cell assemblies by replays helps to reduce instabilities in the large-scale representation of the environment and to reinstate the correct topological structure of the cognitive map.

## THE MODEL

### General Description

The topological model of spatial learning rests on the insight that the hippocampus produces a topological representation of spatial environments and of mnemonic memories—a rough-and-ready framework that is filled with geometric details by other brain regions (Dabaghian, Brandt, & Frank, 2014). This approach, backed up by a growing number of experimental (Alvernhe, Sargolini, & Poucet, 2012; Fenton, Csizmadia, & Muller, 2000; Gothard, Skaggs, & McNaughton, 1996; Knierim, Kudrimoti, & McNaughton, 1998; Leutgeb et al., 2005; Moser, Kropff, & Moser, 2008; Touretzky et al., 2005; Wills, Lever, Cacucci, Burgess, & O’keefe, 2005; Yoganarasimha, Yu, & Knierim, 2006) and computational (Chen, Gomperts, Yamamoto, & Wilson, 2014; Curto & Itskov, 2008; Petri et al., 2014) studies, and allows using a powerful arsenal of methods from algebraic topology, in particular persistent (Carlsson, 2009; Lum et al., 2013; Singh et al., 2008) and zigzag (Carlsson & De Silva, 2010; Carlsson, De Silva, & Morozov, 2009) homology theory techniques, for studying structure and dynamics of the hippocampal map. In particular, the approaches developed in Arai et al. (2014); Babichev, Cheng, & Dabaghian (2016); Babichev, Ji, Mémoli, & Dabaghian (2016); Basso et al. (2016); Dabaghian et al. (2012); Hoffman, Babichev, & Dabaghian (2016) help to explain how the information provided by the individual place cells combines into a large-scale map of the environment, to follow how the topological structure of this map unfolds in time, and to evaluate the contributions made by different physiological parameters into this process. It was demonstrated, for example, that the ensembles of rapidly recycling cell assemblies can sustain stable qualitative maps of space, provided that the network’s rewiring rate is not too high. Otherwise the integrity of the cognitive map may be overwhelmed by topological fluctuations (Babichev & Dabaghian, 2017a, 2017b; Babichev et al., 2018).

**Figure 1.**
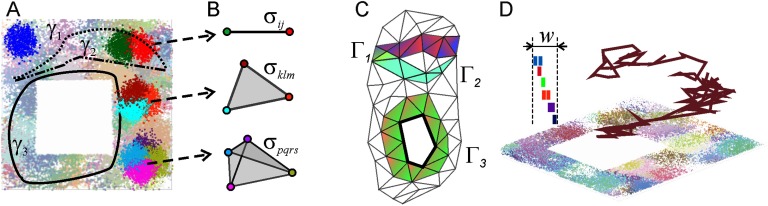
Basic notions of the hippocampal physiology in the context of the topological model. (A) In the following, we simulate rat’s navigation in a square-shaped environment 𝓔 with a hole in the middle. The curves *γ*_1_, *γ*_2_, and *γ*_3_ represent a few short segments of a physical trajectorynavigated by the rat. The place fields—the regions where the corresponding place cells become active—densely cover the environment, forming a place field map *M*_𝓔_. An exemplary place field is represented by a highlighted cluster of blue dots in the top left corner of the environment. The right segment of the environment shows a highlighted pair, a triple and a quadruple of the overlapping place fields; the remaining place fields are dimmed into background. (B) Simplexes that correspond to overlapping place fields: a single vertex corresponds to place field (or a single active cell); a link between two vertexes represents a pair of overlapping place fields (or a pair of coactive cells); three overlapping place fields (or a triple of coactive place cells) correspond to a triangle, and so forth. (C) A collection of simplexes forms a simplicial complex, which schematically represents the net structure of the place field map. Shown is a fragment of a two-dimensional (2D) coactivity complex with simplicial paths Γ_1_, Γ_2_, and Γ_3_ that represent the physical paths *γ*_1_, *γ*_2_, and *γ*_3_ shown on the left. The classes of equivalent simplicial paths describe the topological structure of the coactivity complex: the number of topologically inequivalent, contractible simplicial paths such as Γ_1_ and Γ_2_, defines the number of pieces, *b*_0_, of the coactivity complex (see Methods). The number of topologically inequivalent paths contractible to a one-dimensional (1D) loop defines the number *b*_1_ of holes and so forth (Hatcher, 2002). (D) A schematic representation of a replayed sequence of place cells, shown over the corresponding place fields. The colored ticks in the top left corner schematically represent a sequence of spikes replayed within a short time window *w*.

Mathematically, the method is based on representing the combinations of coactive place cells in a topological framework, as simplexes of a specially designed simplicial complex (Figure 1A and 1B). Each individual simplex *σ* schematically represents a connection (e.g., an overlap) between the place fields encoded by the corresponding place cells’ coactivity. The full set of such simplexes—the coactivity simplicial complex 𝒯—incorporates the entire pool of connections encoded by the place cells in a given environment 𝓔, and hence represents the topological structure of the cognitive map of the navigated space (Best, White, & Minai, 2001; O’Keefe & Dostrovsky, n.d.).

### Topological Structure of the Coactivity Complex

The topological structure of the coactivity complex provides a convenient framework for representing spatial information encoded by the place cells. For example, the combinations of the cells ignited during the rat’s moves along a physical trajectory *γ*, or during a mental replay of such a trajectory, is represented by a “simplicial path”—a chain of simplexes Γ = {*σ*_1_, *σ*_2_, …, *σ*_*k*_} that qualitatively represents the shape of *γ*. A simplicial path that loops onto itself represents a closed physical rout; a pair of topologically equivalent simplicial paths represent two similar physical paths and so forth (Figure 1C).

The net structure of the simplicial paths running through a given simplicial complex 𝒯 can be used to describe its topological shape. Specifically, the number of topologically distinct (counted up to topological equivalence) closed paths that contract to zero-dimensional vertexes—the zeroth Betti number *b*_0_(𝒯)—enumerates the connected components of 𝒯; the number of topologically distinct paths that contract to closed chains of links—the first Betti number *b*_1_(𝒯)—counts its holes and so forth (see (Aleksandrov, 1965; Hatcher, 2002); and Methods).

### Dynamics of the Coactivity Complexes

In practice, the coactivity complexes can be designed to reflect particular physiological properties of the cell assemblies. For example, the time course of the simplexes’ appearance may reflect the dynamics of the cell assemblies’ formation (Babichev & Dabaghian, 2017a, 2017b Babichev et al., 2018; Hoffman et al., 2016), or the details of the place cell activity modulations by the brain waves (Arai et al., 2014; Basso et al., 2016) and so on. In particular, a population of forming and disbanding cell assemblies can be represented by a set of appearing and disappearing simplexes, that is, by a “flickering” coactivity complex 𝓕 studied in Babichev & Dabaghian (2017a, 2017b) and Babichev et al. (2018). There it was demonstrated that if a cell assembly network rewires sufficiently slowly (tens of seconds to a minute timescale), then the “topological shape” of the corresponding coactivity complex remains stable and equivalent to the topology of the simulated environment 𝓔 shown on Figure 1A, as defined by its Betti numbers *b*_*k*_(𝓕) = *b*_*k*_(𝓔) = 1, *k* = 0, 1 (see Methods). Physiologically, this implies that cell assemblies’ turnover at the intermediate and the short memory timescales does not prevent the hippocampal network from producing a lasting representation of space, despite perpetual changes of its functional architecture (Wang et al., 2006).

**Figure 2.**
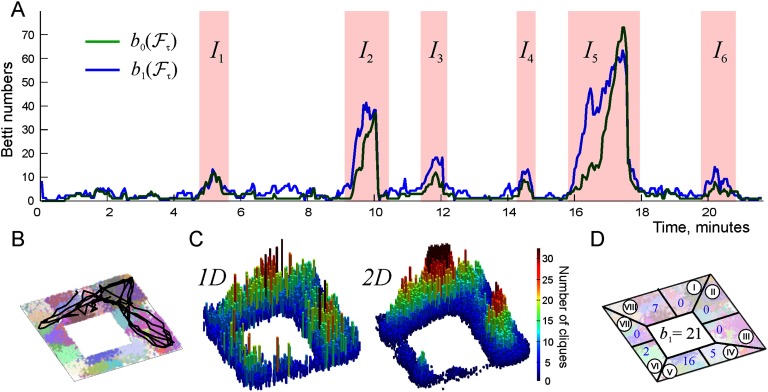
Topological fluctuations in a rapidly decaying coactivity complex in absence of replays. (A) The green and the blue lines show, respectively, the zeroth and the first Betti numbers, *b*_0_(𝓕_*τ*_) and *b*_1_(𝓕_*τ*_) (see Methods), as functions of time. For most of the time, both Betti numbers remain small, 〈*b*_0_(𝓕_*τ*_)〉 ≈ 2.5 ± 2.1 and 〈*b*_1_(𝓕_*τ*_)〉 ≈ 2.8 ± 2.2, indicating a few disconnected fragments of the coactivity complex 𝓕_*τ*_ and a few spurious holes in them. The rapid increase of the Betti numbers during short “instability intervals” *I*_1_, …, *I*_6_ (highlighted by the pink background) indicate periods of strong topological fluctuations in 𝓕_*τ*_. (B) A segment of the simulated trajectory taken between the 16th and 18th minute shows that the animal spends time before and during the instability period *I*_5_ in a particular segment of the arena. During this time, the connections over the unvisited segments of 𝓔 start to decay (here the connections’ mean proper lifetime is *τ* = 50 s), as a result of which the coactivity complex 𝓕_*τ*_ fractures into a large number of disconnected pieces riddled in holes, which explains the splash of *b*_0_(𝓕_*τ*_) and *b*_1_(𝓕_*τ*_). (C) Spatial histograms of the links (i.e., centers of the pairwise place field overlaps, left panel) and of the three-vertex simplexes (i.e., centers of triple place field overlaps, right panel) present in 𝓕_*τ*_ during the instability period *I*_5_. The simplexes concentrate over the northeast corner of the environment, whereas the populations of simplexes over the south and the southwestern parts thin out. (D) The “local” Betti number *b*_1_ (blue numerals) computed separately for the eight sectors of the environment (circled Roman numerals) indicate that the holes emerge in all the “abandoned” parts, for example, sector IV contains 5 holes and sector V contains 16 holes, and so forth. The global Betti number computed at about 16th minute for the entire complex, *b*_1_(𝓕_*τ*_) = 21, is shown in the middle.

In particular, the model (Babichev et al., 2018) predicts that cell assembly network produces a stable topological map if the connections’ mean lifetime exceeds *τ* ≥ 150–200 s, which corresponds to the Hebbian plasticity timescale (Billeh et al., 2014; Goldman-Rakic, 1995; Hiratani & Fukai, 2014; Russo & Durstewitz, 2017; Zenke & Gerstner, 2017). For noticeably shorter *τ*, the topological fluctuations in the simulated hippocampal map are too strong and a stable representation of the environment fails to form. For example, in the case of the place field map shown on Figure 2A, the connections’ proper lifetime is about *τ* = 50 s and the corresponding coactivity complex is unstable: its Betti numbers frequently exceed the physical values (*b*_*k*_(𝓕) > *b*_*k*_(𝓔)), implying that 𝓕 may split into several disconnected pieces, each one of which may contain transient gaps, holes, and other topological defects that do not correspond to the physical features of the environment.

For most of the time, these defects are scarce (*b*_*k*_(𝓕) < 5, Figure 2A) and may be viewed as topological irregularities that briefly disrupt otherwise functional cognitive map. Indeed, from the physiological perspective, it may be unreasonable to assume that biological cognitive maps never produce topological inconsistencies—in fact, admitting small fluctuations in a qualitatively correct representation of space may be biologically more effective than spending time and resources on acquiring a precise and static connectivity map, especially in dynamically changing environments. However, during certain periods, the topological fluctuations may become excessive, indicating the overall instability of the cognitive map. The origin of such occurrences is clear: if, for example, the animal spends too much time in particular parts of the environment, then the parts of 𝓕 that represent the unvisited segments of space begin to deteriorate, leaving behind holes and disconnected fragments (Figure 2B–2D). Outside of these “instability periods,” when the rat regularly visits all segments of the environment, most place cells fire recurrently, thus preventing the coactivity complex 𝓕 from deteriorating.

Although this description does not account for the full physiological complexity of synaptic and structural plasticity processes in the cell assembly network, it allows building a qualitative model that connects the animal’s behavior, the parameters describing deterioration of the hippocampal network’s functional architecture and the large-scale topological properties of the cognitive map. This, in turn, provides a context for testing the effects produced by the place cell replays, for example, their alleged role in acquiring and stabilizing memories by strengthening the connections in parahippocampal networks (Colgin, 2016; Sadowski et al., 2011, 2016). To test these hypotheses, we adopted the topological model (Babichev et al., 2018) so that the decaying connections in the simulated hippocampal cell assemblies can be (re)established not only by the place cell activity during physical navigation but also by the endogenous activity of the hippocampal network, and studied the effect of the latter on the structure of the hippocampal map, as outlined below.

### Implementation of the Coactivity Complexes

Implementation of the coactivity complexes is based on a classical model of the hippocampal network, in which place cells *c*_*i*_ are represented as vertexes *v*_*i*_ of a “cognitive graph” 𝒢, while the connections between pairs of coactive cells are represented by the links, *ς*_*ij*_ = [*v*_*i*_, *v*_*j*_] of this graph (Babichev, Cheng, & Dabaghian, 2016; Burgess & O’keefe, 1996; Muller, Stead, & Pach, 1996). The assemblies of place cells *ς* = [*c*_1_, *c*_2_, …, *c*_*n*_]—the “graphs of synaptically interconnected excitatory neurons,” according to Buzsáki (2010)—then correspond to fully interconnected subgraphs of 𝒢, that is, to its maximal cliques (Babichev, Cheng, & Dabaghian, 2016; Babichev et al., 2016; Hoffman et al., 2016). Since each clique *ς*, as a combinatorial object, can be viewed as a simplex spanned by the same set of vertexes (see Supplemental Figure 6 in Basso et al., 2016), the collection of cliques of the graph 𝒢 defines a clique simplicial complex (Jonsson, 2008), which proves to be one of the most successful implementations of the coactivity complex. In previous studies (Babichev, Cheng, & Dabaghian, 2016; Babichev et al., 2016; Basso et al., 2016; Hoffman et al., 2016), we demonstrated that in absence of decay (*τ* = ∞), such a complex 𝒯 effectively accumulates information about place cell coactivity at various timescales, capturing the correct topology of planar and voluminous environments. If the decay of the connections is taken into account (*τ* < ∞), then the topology of the “flickering” coactivity complex 𝓕 remains stable for sufficiently small rates, but if *τ* becomes too small, the topology of 𝓕 may degrade. A question arises, whether the replays can slow down its deterioration, as the biological considerations suggest.

### Dynamics of the Coactivity Graph

Physiologically, place cell spiking is synchronized with the components of the extracellular local field potential—the so-called brain waves that also define the timescale of place cell coactivity (Buzsaki, 2006). Specifically, two or more place cells are considered coactive if they fire spikes within two consecutive *θ*-cycles—approximately 150–250 ms interval (Mizuseki, Sirota, Pastalkova, & Buzsáki, 2009)—a value that is also suggested by theoretical studies (Arai et al., 2014). In the following, this period will define the shortest timescale at which the functional connectivity of the simulated hippocampal network can change. For example, a new link *ς*_*ij*_ = [*v*_*i*_, *v*_*j*_] in the coactivity graph will appear, if a coactivity of the cells *c*_*i*_ and *c*_*j*_ was detected during a particular 2*θ* period. In absence of coactivity, the links can also disappear with probability

$$p_{0}(t)=\frac{1}{\tau }e^{-t/\tau },$$ (1)

where *t* is the time measured from the moment of last spiking of both cells *c*_*i*_ and *c*_*j*_ and the parameter *τ* defines the mean lifetime of the synaptic connections in the cell assembly network. In the following, *τ* will be the only parameter that describes the deterioration of the synaptic connections within the cell assemblies (Babichev et al., 2018). We will therefore use the notations 𝒢_*τ*_ and 𝓕_*τ*_ to refer, respectively, to the flickering coactivity graph with decaying connections and to the resulting flickering coactivity complex with decaying simplexes.

### Replays of Place Cell Sequences

Replays of place cell sequences may in general represent both spatial and nonspatial memories. In the following, we simulate only spatial replays by constructing simplicial paths that represent previously navigated trajectories. Specifically, we select chains of connections that appeared in the coactivity graph 𝒢_*τ*_ at the initial stages of navigation and reactivate them at the later replay times *t*_*r*_, *r* = 1, 2, …, *N*_*r*_ (Kudrimoti, Barnes, & McNaughton, 1999; O’Neill, Senior, & Csicsvari, 2006). To replay a trajectory originating at a given timestep *t*_*i*_, we randomly select a coactivity link $\varsigma_{kl}^{\left(i\right)}$ ∈ 𝒢_*τ*_(*t*_*i*_) that is active within that time window; this link then gives rise to a sequence of joined links, randomly selected among the ones that activate at the consecutive time steps, $\varsigma_{lm}^{\left(i+1\right)}$, $\varsigma_{mn}^{\left(i+2\right)}$, …. Since there are typically several active links at every moment, this procedure allows generating a large number of replay trajectories. The physiological duration of replays—typically about 100–200 ms (Colgin, 2016)—roughly corresponds to the coactivity window widths, that is, to the timesteps in which the coactivity graph evolves; we therefore “inject” the activated links into a particular coactivity window *t*_*r*_ in order to simulate rapid replays.

After a simplicial trajectory is replayed, the injected links begin to decay and to (re)activate in the course of the animal’s moves across the environment, just as the rest of the links. Most of these “reactivated” links simply rejuvenate the existing connections in 𝒢_*τ*_. However, some injections instantaneously reinstate decayed connections and produce an additional population of higher order cliques, which affect the topological properties of the coactivity complex 𝓕_*τ*_, and hence—according to the model—of the cognitive map. As mentioned previously, hippocampal replays are believed to enable spatial learning by stimulating inactive connections, by slowing down their decay, and by reinforcing cell assemblies’ stability (Carr et al., 2011; Ego-Stengel & Wilson, 2010; Girardeau et al., 2009; Girardeau & Zugaro, 2011; Jadhav et al., 2012; Sadowski et al., 2016). In the model’s terms, this hypothesis translates as follows: the additional influx of rejuvenated simplexes provided by the replays should qualitatively improve the topological structure of the flickering coactivity complex, slow down deterioration of its simplexes, suppress its topological defects, and, in general, help to sustain its topological integrity. In the following, we test this hypothesis by simulating different patterns of the place cell reactivations and quantifying the effect that this produces on the simulated cognitive map.

## RESULTS

### Initial Testing

**Figure 3.**
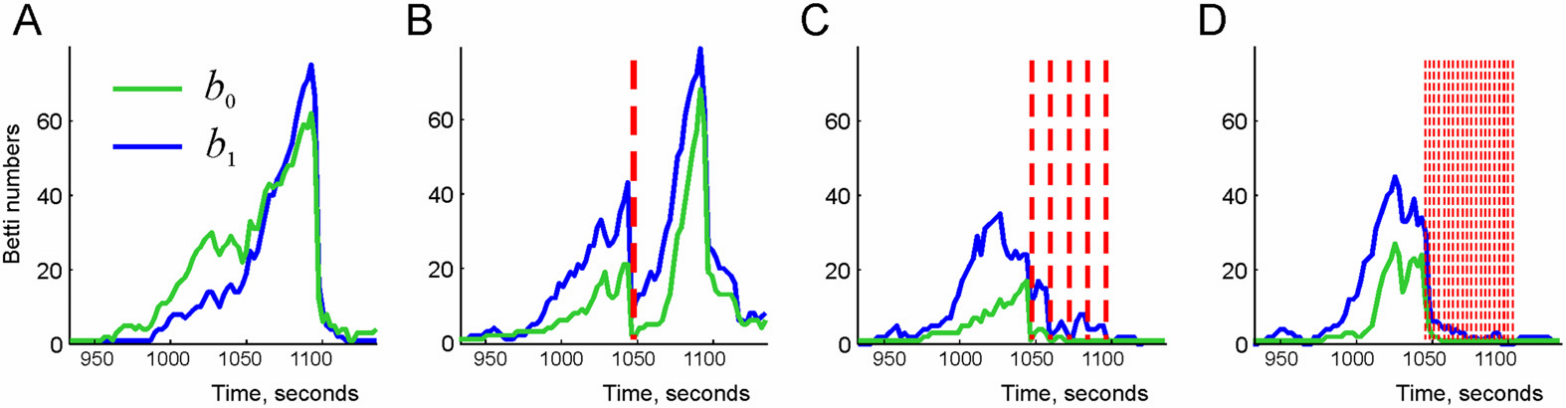
Suppressing topological fluctuations by reactivation of simplexes. (A) During the instability period *I*_5_ (approximately between 15.5 and 18.5 min) the topological fluctuations in the coactivity complex become very strong, with the Betti numbers soaring at *b*_0_(𝓕_*τ*_) ≈ 65 and *b*_1_(𝓕_*τ*_) ≈ 75. (B) If all the reactivated links are injected into the coactivity complex at once, at a moment preceding the peak of the Betti numbers (marked by a vertical red dashed line), the fluctuations in the coactivity complex are immediately suppressed. However, as the connection decay takes over, the fluctuations kick back, reaching the original high values in under a minute. (C) Five consecutive replays, marked by five vertical red dashed lines, produce a more lasting effect, reducing the Betti numbers to smaller values 〈*b*_0_(𝓕_*τ*_)〉 ≈ 3 and 〈*b*_1_(𝓕_*τ*_)〉 ≈ 7 over the remainder of the instability period. (D) More frequent replays (once every 2.5 s, vertical dashed lines) nearly suppress the topological fluctuations, producing the average values 〈*b*_0_(𝓕_*τ*_)〉 ≈ 1.2 and 〈*b*_1_(𝓕_*τ*_)〉 ≈ 3, that is, leaving only a couple of spurious loops in 𝓕_*τ*_.

The effect produced by the replays on the cognitive map depends on the parameters of the model: the selection of replayed trajectories, the injection times, the frequency of the replays, and so forth. To start the simulations, we selected *N*_*s*_ = 80 different replay sequences originating at *N*_*i*_ = 25 moments of time, *t*_*i*_, *i* = 1, …, *N*_*i*_, between 20 and 200 s of navigation (the initial interval *I*_*init*_). During this period, the trajectory covers the arena more or less uniformly: a typical 25-s-long segment of a trajectory extends across the entire environment and contains on average about *l*_*s*_ = 100 links (Supporting Information Figure S1). As a result, the corresponding simplicial paths traverse the full coactivity complex 𝓕_*τ*_, and one would expect that replaying these paths should help to suppress the topological defects in 𝓕. To verify this prediction, we replayed the resulting pool of 𝒢-link sequences within the main instability period *I*_5_ by using different approaches and tested whether this can suppress the topological fluctuation (Figure 3A).

In the first scenario, all replay chains were injected into the connectivity graph 𝒢_*τ*_ at once, in the middle of the instability period *I*_5_ (Figure 3B). As a result of such a “massive” instantaneous replay, the topological fluctuations are initially suppressed but then they quickly rebound, producing about the same number of spurious 0*D* loops (i.e., the cognitive map remains as fragmented as before) and an even higher number of 1*D* loops that mark spurious holes in the cognitive map (Supporting Information Movie 1). In other words, our model suggests that a single “memory flash” fails to correct the deteriorating memory map even at a short timescale, which suggests that more regular replay patterns are required.

Indeed, if the same set of replay sequences is uniformly distributed into *N*_*r*_ = 5 consecutive groups inside the instability period *I*_5_ (one group per 36 s, *N*_*s*_/*N*_*r*_ = 16 chains of links each), then the topological fluctuations in the coactivity complex 𝓕_*τ*_ subside more and over a longer period (see Figure 3C and Supporting Information Movie 2). If the replays are produced even more frequently (every 9 s, i.e., about 20 replays total, *N*_*s*_/*N*_*r*_ ≈ 4 chains of links injected per replay) then the topological fluctuations in *I*_5_ are essentially fully suppressed over the entire environment (Figure 3D, Supporting Information Movie 3).

One can draw two principal observations from these results: first, that spontaneous reactivation of connections at the physiological timescale can qualitatively alter the topological structure of the flickering coactivity complex, and, second, that the temporal pattern of replays plays a key role in suppressing the topological fluctuations in the cognitive map.

### Implementation of the Replays

Electrophysiological data shows that the frequency of the replays ranges between 0.1 Hz in active navigation to 0.4 Hz in quiescent states and 4 Hz during sleep (Colgin, 2016; Jadhav et al., 2012; O’Neill et al., 2006; Sadowski et al., 2016). Since we model spatial learning taking place during active navigation, we implemented replays at the maximal rate of 0.4 Hz, which corresponds to no more than one replay event over 10 consecutive coactivity intervals. Second, we took into account the fact that, in complex environments, the hippocampus may replay a few sequences simultaneously. For example, on the *Y*-track (O’Neill et al., 2006) two simultaneous replay sequences can represent the two prongs of the *Y*. In open environments, there may be more simultaneously replayed sequences; however, we used the most conservative estimate and replayed two different sequences at each replay moment *t*_*r*_.

**Figure 4.**
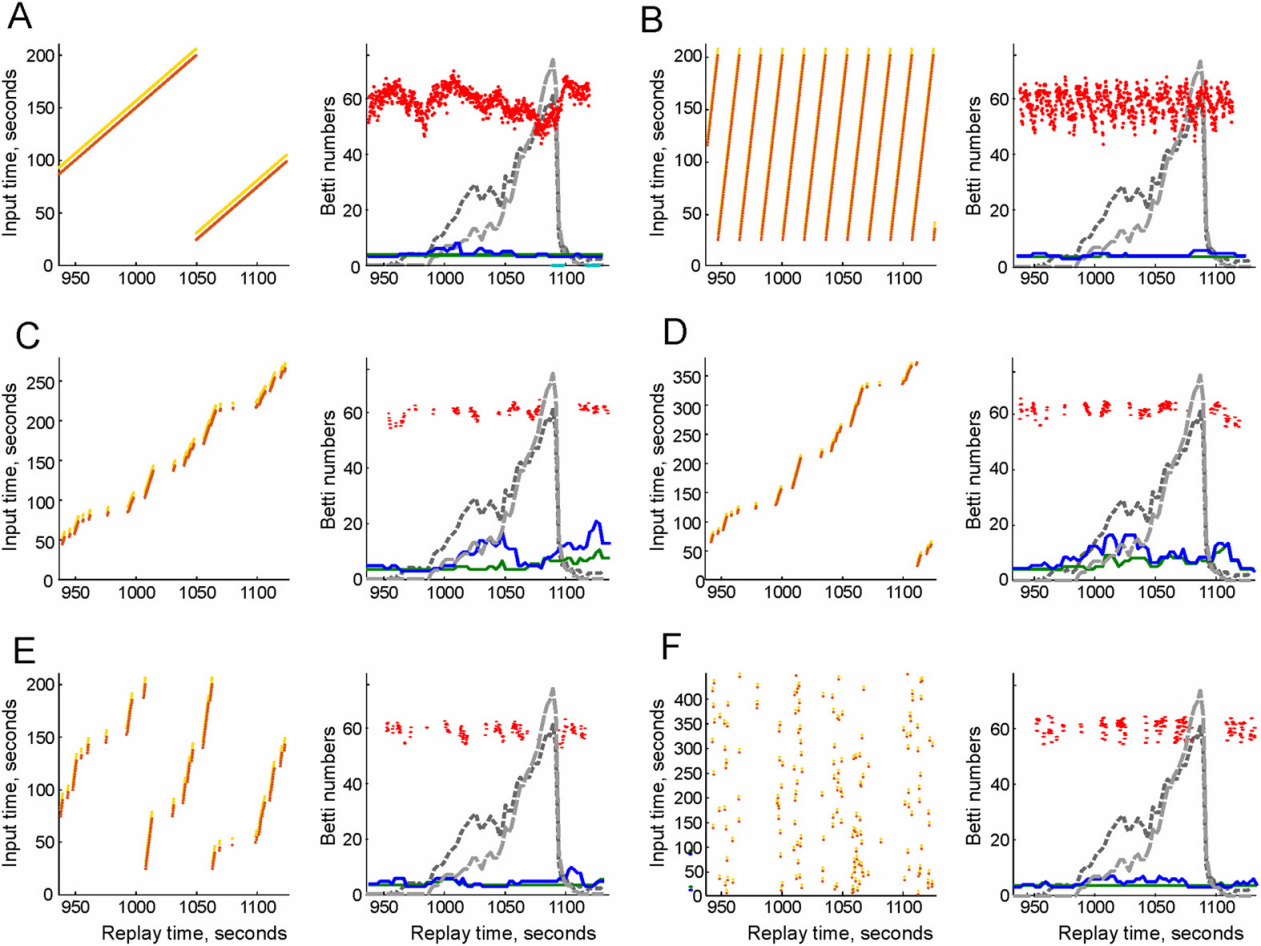
Suppressing the topological fluctuations by replays. In all cases, the injection diagram on the left relates the times when the place cell sequences were originally produced (vertical axis) to the times when they are replayed (horizontal axis). Each yellow and brown dot corresponds to a replayed sequence. On the right panel, the replay times are marked by red dots, with the vertical scatter proportional to the simulated speed of the animal. The resulting zeroth Betti number (*b*_0_(𝓕_*τ*_), green line) and the first Betti number (*b*_1_(𝓕_*τ*_), blue line) are shown in the foreground, and the original, unstable Betti numbers (without replays) are shown in the background (dark and light dashed gray lines, respectively). (A) A simple translational replay of a couple of sequences repeated over a 3-min period (between 20 and 200 s), with a Δ*T* = 14-min delay. The zeroth Betti number regains its physical value, *b*_0_(𝓕_*τ*_) = *b*_0_(𝓔) = 1, indicating that cognitive map reconnects into one piece. The first Betti number fluctuates near the physical value *b*_1_(𝓕_*τ*_) = 1.5 ± 2.2, indicating that nearly all spurious holes are closed. (B) Compressed replay: a 3-min period is replayed repeatedly over several consecutive 20-s intervals. Here the zeroth Betti number remains correct, *b*_0_(𝓕_*τ*_) = 1, and the fluctuations of the first Betti number reduce farther, *b*_1_(𝓕_*τ*_) = 1.2 ± 1. (C) Modulating the replay times by slow move periods (*v* < 15 cm/s) produces sparser replays. As a result, the topological fluctuations in the case of uncompressed, speed-modulated delayed replays increase, *b*_0_(𝓕_*τ*_) = 4.2 ± 1.9, *b*_1_(𝓕_*τ*_) = 8.2 ± 3.4. (D) Small compressions (up to 300 s replayed over ∼ 150 second period, same delay) may intensify the fluctuations: *b*_0_(𝓕_*τ*_) = 5.9 ± 2.4, *b*_1_(𝓕_*τ*_) = 9.2 ± 4.1. (E) Further compression of the replays improves the results, *b*_0_(𝓕_*τ*_) = 1.1 ± 1.4, *b*_1_(𝓕_*τ*_) = 1.7 ± 2, although the variations of *b*_1_(𝓕_*τ*_) remain high compared with the cases in which the replays are not modulated by the speed. (F) Random replays reduce the fluctuations even further: the coactivity complex acquires the correct zeroth Betti number *b*_0_(𝓕_*τ*_) = 1 (the map becomes connected), producing occasional spurious loops, *b*_1_ = 1.4 ± 1.2, that is, occasional topological irregularities.

In the simplest scenario, we injected pairs of sequences into the coactivity graph 𝒢_*τ*_ with a constant delay of about Δ*T* = *t*_*i*_ − *t*_*r*_ ≈ 14 min after their physical onset, which placed them inside of the instability period *I*_5_ (Figure 4A). In response, the topological fluctuations in the coactivity complex 𝓕_*τ*_ significantly diminished. In fact, the zeroth Betti number (the number of the disconnected components) regained its physical value *b*_0_(𝓕_*τ*_) = 1, indicating that the replays helped to pull the fragments of the cognitive map together into a single connected piece. The first Betti number (the number of holes) remains on average close to its physical value, 〈*b*_1_(𝓕_*τ*_)〉 = 1.5, exhibiting occasional fluctuations, Δ*b*_1_ = ±2.2.

As mentioned above, the occasional islets separating from the main body of the simplicial complex or a few small holes appearing in it for a short period should be viewed as topological irregularities rather than signs of topological instability. We therefore base the following discussion on addressing only the qualitative differences produced by the replays on the topology of the cognitive map: whether replays can prevent fracturing of the complex into multiple pieces and rapid proliferation of spurious loops in all dimensions. From such perspective, our results demonstrate that translational replays at a physiological rate can effectively restore the correct topological shape of the cognitive map, which illustrates functional importance of the replay activity.

Since the replays are generated by the endogenous activity of the hippocampal network, the relative temporal order of the replayed sequences can be altered, that is, the replay times *t*_*r*_ can be spread wider or denser than their “physical” origination times *t*_*i*_. The effect of the replays will be, respectively, weaker or stronger than in the case of translational delay because of the corresponding changes of the sheer number of the reactivated links. However, one can factor out the direct contribution of the replays’ volume and study more subtle effects produced specifically by the replay’s temporal organization. To this end, we split the replay period *I*_5_ into a set of *N*_*R*_ shorter subintervals, $I_{5}^{1}$, $I_{5}^{2}$, … $I_{5}^{N_{R}}$, and then replayed the sequences of links originating from the initial 3-min interval *I*_*init*_ within each subinterval $I_{5}^{n}$, *n* = 1, 2, …, *N*_*R*_. Since only two sequences are replayed within every coactivity window, the total number of the (re)activated sequences remains the same as in the delayed replay case, even though the source interval *I*_*init*_ is compressed *N*_*R*_-fold in time. Thus, the difference between the effects produced by the “compressed” replays will be due solely to the differences in their temporal reorganizations.

The results illustrated in Figure 4B demonstrate that the compressed replays suppress the topological fluctuations more effectively. For example, the repeated replay in a sequence of 20-s intervals (*N*_*R*_ = 9 fold compression) not only restores the correct value of the zeroth Betti number, *b*_0_(𝓕_*τ*_) = 1, but also drives the average number of noncontractible simplicial loops close to physical value, 〈*b*_1_(𝓕_*τ*_)〉 = 1.2 ± 1. In other words, 𝓕_*τ*_ almost regains its topologically correct shape, with an occasional spurious hole appearing for less than a second. Physiologically, these results suggest that time-compressed, repetitive “perusing” through memory sequences helps to prevent deterioration of global memory frameworks better than simple “orderly” recalls.

### Speed Modulation of the Replays

Since replays are mostly observed during quiescent periods and slow moves (O’Neill et al., 2006; O’Neill, Senior, Allen, Huxter, & Csicsvari, 2008), we studied whether such “low-speed” replays will suffice for suppressing the topological fluctuations in the cognitive map. Specifically, we identified the periods when the speed of the animal falls below 15 cm/sec (which, in our simulations happens during 14% of time (see Supporting Information Figure 3 and Koene & Hasselmo, 2008; Nádasdy, Hirase, Czurkó, Csicsvari, & Buzsáki, 1999), and replayed the place cell sequences only during these periods.

It turns out that although the resulting slow motion replays can stabilize the topological structure of the simulated cognitive map, the effect strongly depends on their temporal organization. Specifically, in the simple delayed replay scenario, the topological fluctuations remain significantly higher than without speed modulation (Figure 4C and Movie 4, Supporting Information). On average, the coactivity complex contains about a dozen spurious loops: it remains split in a few pieces, 〈*b*_0_(𝓕_*τ*_)〉 = 4.2, that together contain 〈*b*_1_(𝓕_*τ*_)〉 = 8.2 holes on average. This is a natural result—one would expect that speed restrictions will diminish the number of the injected active connections and hence that 𝓕_*τ*_ will degrade more. A slightly compressed replay (4 min of activity replayed over 3-min period) does not improve the result: both the number of disconnected components and the number of holes in them increase 〈*b*_0_(𝓕_*τ*_)〉 = 5.9, 〈*b*_1_(𝓕_*τ*_)〉 = 9.2 (Figure 4D). However, if the replays are compressed further, the average number of disconnected components is significantly reduced: for the threefold compression shown on Figure 4E, the mean values are 〈*b*_0_(𝓕_*τ*_)〉 = 1.1 and 〈*b*_1_(𝓕_*τ*_)〉 = 1.7, that is, the encoded map approaches the quality of the maps produced with unrestricted replays.

The effectiveness of the latter scenario can be explained by noticing that replay compression brings the activities that are widely spread in physical time into close temporal vicinities during the replays. In other words, in compressed replays, a wider variety of connections is activated at each *t*_*r*_: the real-time separation between activity patterns shrinks. This helps to reduce or eliminate the temporal “lacunas” in place cell coactivity across the entire hippocampal network and hence to prevent spontaneous deterioration of its parts. In physiological terms, this implies that the compressed replays of the place cell patterns are less constrained by the physical temporal scale of the rat’s navigational experiences, which leads to a more even activation of the connections in the network and helps to prevent the memory map’s fragmentation.

**Figure 5.**
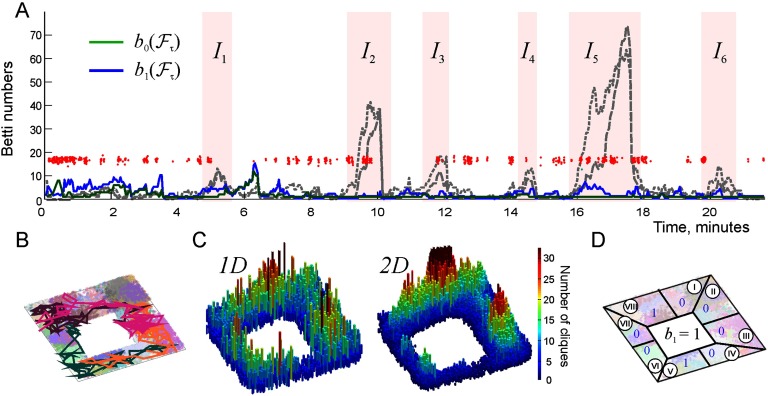
Replays suppress topological fluctuations over the entire navigation period. (A) The time dependence of the Betti numbers *b*_0_(𝓕_*τ*_) and *b*_1_(𝓕_*τ*_) (the green and the blue line respectively) in presence of the replays. The topological fluctuations that previously overwhelmed the map during the “instability intervals” (shown in the background by two dashed gray lines, see Figure 2A) are now nearly fully suppressed. The replay moments, *t*_*r*_, marked by the red dots, are modulated by speed, *v* < 15 cm/s. (B) Several examples of replayed trajectories over the navigated environment are shown in different colors (see also Supporting Information Figure 1). (C) Spatial histograms of the centers of the links (left panel) and of the three-vertex simplexes (right panel) during the instability period *I*_5_ in presence of the replays. The populations of simplexes over the south and the southwestern parts of the environment in presence of the replays have increased compared to the case shown on Figure 2C, which suppresses spurious topological loops in the coactivity complex. (D) The Betti numbers *b*_1_ computed for the eight sectors of the environment are also significantly reduced, indicating that the topological fluctuations are suppressed both locally and globally. The deviations of *b*_1_ from 0 in the sectors V and VIII are due to boundary effects at the sector’s edges that do not affect the global value *b*_1_(𝓕_*τ*_) = 1. All zeroth Betti numbers, both local and global, assume correct values *b*_0_ = 1 and are not shown.

To test this idea, we amplified this effect by shuffling the order of the replayed sequences and by randomizing the injection diagram, thus enforcing a nearly uniform pattern of injected activity across the simulated population of cell assemblies. This indeed proved to be the most effective replay strategy: as shown on Figure 4F, such replay patterns restore the topological shape of the coactivity complex, allowing only occasional holes: 〈*b*_0_(𝓕_*τ*_)〉 = 1, 〈*b*_1_(𝓕_*τ*_)〉 ≈ 1.4 ± 1.2 (Supporting Information Movie 5). Thus, the model suggests that “reshuffling” the temporal sequence of memory replays helps to sustain memory framework better than orderly recollections, occurring in natural past-to-future succession. The effect of random replays of the place cell sequences over the entire simulated navigation period shown in Figure 5 clearly illustrates the importance of replays for rapid encoding of topological maps: the fluctuations in the cognitive map are uniformly suppressed (for the original values of the Betti numbers during all six instability periods without replays see Table 1 in Methods).

**Table 1.** Betti number statistics for the six instability periods (Figures 2) without replays: the mean ${\overset{-}{b}}_{k}$ and the variance Δ*b*_*k*_, for *k* = 0, 1.

**Instability period**	${\overset{-}{b}}_{0}$	**Δ*b*_0_**	${\overset{-}{b}}_{1}$	**Δ*b*_1_**
*I*_1_	7.7	4.2	8.2	4.3
*I*_2_	17.9	9.8	28.7	11.1
*I*_3_	7.1	3.8	14.2	3.8
*I*_4_	5.5	3.4	8.9	4.4
*I*_5_	27.8	19.8	34.9	21.2
*I*_6_	5.7	3.8	7.7	3.1

## DISCUSSION

The model discussed above suggests that replays of place cell activity help to learn and to sustain the topological structure of the cognitive map. The physiological accuracy of the replay simulation can be increased ad infinitum, by incorporating more and more parameters into the model. In this study we use only a few basic properties of the replays, which, however, capture several key functional aspects of the replay activity. First, the model implements an effective feedback loop, in which the onset of topological instabilities in the flickering coactivity complex 𝓕_*τ*_ triggers the replays that restore its integrity. Indeed, the cell assemblies’ (and the corresponding simplexes’) decays intensify as the animal’s exploratory movements slow down and visits to particular segments of the environment become less frequent. On the other hand, low-speed periods define temporal windows during which the simulated replays are injected into the network, which work to suppress the topological instabilities. Second, the model allows controlling the replays’ temporal organization independently from the other parameters or neuronal activity and exploring the replays’ contribution into acquiring and stabilizing the cognitive maps. The results demonstrate that in order to strengthen the decaying connections in the hippocampal network effectively, the replays must (1) be produced at a sufficiently high rate that falls within the physiological range and (2) distribute without temporal clustering, in a semi-random order.

An important aspect of the obtained results is a separation of the timescales at which different types of topological information is processed. On the one hand, rapid turnover of the information about local connectivity at the working memory timescale is represented by quick recycling of the cell assemblies and rapid spontaneous replays of the learned sequences. On the other hand, the large-scale topological structures of the cognitive map, described by the instantaneous homological characteristics of the coactivity complex, emerge at the intermediate memory timescale. Thus, the model suggests that the characteristic timescale of the topological loops’ dynamics is by an order of magnitude larger than the timescale of fluctuations at the cell assembly level. This observation provides a functional perspective on the role played by the place cell replays in learning: by reducing the fluctuations, replays help separating the fast and the slow information processing timescales and hence to extract stable topological information that can be used to build a long-term, qualitative representation of the environment. This separation of timescales corroborates with the well-known observation that transient information is rapidly processed in the hippocampus and then the resulting memories are consolidated and stored in the cortical areas, but at slower timescales and for longer periods.

## METHODS

### Topological Glossary

For the reader’s convenience, we briefly outline the key topological terms and concepts used in this paper.

▪  *An abstract simplex* of order *d* is a set of (*d* + 1) elements, for example, a set of coactive cells, *σ*^(*d*)^ = [*c*_*i*_0__, *c*_*i*_1__, …, *c*_*i*_*d*__] or a set of place fields, *σ*^(*d*)^ = [*υ*_*j*_0__, *υ*_*j*_1__, …, *υ*_*j*_*k*__]. The subsets of *σ*^(*d*)^ are its *subsimplexes*. Subsimplexes of maximal dimensionality (*d* − 1) are referred to as *facets* of *σ*^(*d*)^.
▪  *An abstract simplicial complex* Σ is a family of abstract simplexes closed under the overlap relation: a nonempty overlap of any two simplexes $\sigma_{1}^{\left(d_{1}\right)}$ ∈ Σ and $\sigma_{2}^{\left(d_{2}\right)}$ ∈ Σ is a subsimplex of both $\sigma_{1}^{\left(d_{1}\right)}$ and $\sigma_{2}^{\left(d_{2}\right)}$.
▪  Geometrically, simplexes can be visualized as *d*-dimensional polytopes: *σ*^(0)^ as a point, *σ*^(1)^ as a line segment, *σ*^(2)^ as a triangle, *σ*^(3)^ as a tetrahedron, and so forth. The corresponding *geometric simplicial complexes* are multidimensional polyhedra that have a shape and a *structure* that does not change with simplex deformations, for example, disconnected components, holes, cavities of different dimensionality, and so on. This structure, commonly referred to as *topological* (Aleksandrov, 1965), is identical in a geometric simplicial complex to and in the abstract complex built over the vertexes of the geometric simplexes. Thus, abstract simplicial complexes may be viewed as structural *representations* of the conventional geometric shapes.
▪  Topological properties of the simplicial complexes are established based on algebraic analyses of chains, cycles and boundaries.

*A chain* *α*^(*d*)^ is a formal combination *d*-dimensional simplexes with coefficients from an algebraic ring or a field. Intuitively, they can be viewed, for example, as the simplicial paths described in The Model section. Such combinations permit algebraic operations: they can be added, subtracted, and multiplied by a common factor, and so forth. As a result, the set of all chains of a given simplicial complex, *C*(Σ), also forms an algebraic entity, for example, if the chains’ coefficients form to a field, then *C*(*Σ*) forms a vector space.

*A boundary* of a chain, ∂*α*^(*d*)^, is a formal combination of all the facets of the *α*-chain, with the coefficients inherited from *α* and alternated so that the boundary of ∂*α*^(*d*)^ vanishes, ∂^2^*α*^(*d*)^ = 0. This universal topological principle—boundary of a boundary is a null set—can be illustrated on countless examples, for example, by noticing that the external surface of a triangular pyramid *σ*^(3)^—its geometric boundary—has no boundary itself.

*Cycles* generalize the previous example—a generic cycle *z* is a chain without a boundary, ∂*z* = 0. Intuitively, cycles correspond to agglomerates of simplexes (e.g., simplicial paths) that loop around holes and cavities of the corresponding dimension. Note however, that although all boundaries are cycles, not all cycles are boundaries.

▪  *Homologies* are two cycles, *z*_1_ and *z*_2_, that are *equivalent*, or *homologous*, if they differ by a boundary chain. The set of equivalent cycles forms a *homology class*. If the chain coefficients come from a field, then the homology classes of *d*-dimensional cycles form a vector space H_*d*_(Σ). The dimensionality of this vector space is the *d*-th *Betti number* of the simplicial complex Σ, *b*_*d*_(Σ) = dim H_*k*_(Σ), which counts the number of independent *d*-dimensional holes in Σ.
▪  *Flickering complexes* 𝓕(*t*) consist of simplexes that may disappear or (re)appear, so that the complex as a whole may grow or shrink from one moment to another (see Figure 2 in Babichev et al., 2018),
  $$F(t_{1})\subseteq F(t_{2})\subseteq F(t_{3})⊇F(t_{4})\subseteq F(t_{5})⊇\ldots .$$
  Computing the corresponding Betti numbers, *b*_*k*_(𝓕(*t*)), requires a special technique—*Zigzag persistent homology* theory that allows tracking cycles in 𝓕 on moment-to-moment basis (Babichev et al., 2018; Carlsson & De Silva, 2010; Carlsson et al., 2009).
▪  *A clique* in a graph *G* is a set of fully interconnected vertices, that is, a complete subgraph of *G*. Combinatorially, cliques have the same key property as the abstract simplexes: any subcollection of vertices in a clique is fully interconnected. Hence a nonempty overlap of two cliques *ς* and *ς*′ is a subclique in both *ς* and *ς*′, which implies that cliques may be formally viewed as abstract simplexes and a collection of cliques in a given graph *G* produces its *clique simplicial complex* Σ(*G*) (Jonsson, 2008). In particular, the *clique coactivity complexes* 𝒯_*ς*_ is induced from the *coactivity graphs* 𝒢 (Babichev et al., 2016; Basso et al., 2016; Hoffman et al., 2016) and the flickering clique complexes 𝓕_*τ*_ are constructed using coactivity graph with flickering connections 𝒢_*τ*_, (Babichev & Dabaghian, 2017a, 2017b; Babichev et al., 2018). Note however, that the topological analyses address the topology of the coactivity complexes, rather than the network topology of 𝒢.

### Spike Simulations

The environment shown on Figure 1A is simulated after typical arenas used in typical electrophysiological experiments. Over the navigation period *T*_*tot*_ = 30 min, the trajectory covers the environment uniformly. The maximal speed of the simulated movements is *v*_max_ = 50 cm/s, with the mean value $\overset{-}{v}$ = 25 cm/s. The firing rate of a place cell *c* is defined by

$$\lambda_{c}(r)=f_{c}e^{-\frac{{\left(r-r_{c}\right)}^{2}}{2s_{c}^{2}}}$$

where *f*_*c*_ is the maximal firing rate and *s*_*c*_ defines the size of the place field centered at *r*_*c*_ (Barbieri et al., 2004). In addition, spiking is modulated by the *θ*-oscillations—a basic cycle of the extracellular local field potential in the hippocampus, with the frequency of about 8 Hz (Arai et al., 2014; Huxter, Senior, Allen, & Csicsvari, 2008; Mizuseki et al., 2009). The simulated ensemble contains *N*_*c*_ = 300 virtual place cells, with the typical maximal firing rate *f* = 14 Hz and the typical place field size *s* = 20 cm.

### The Statistics of the Betti Numbers

The values during instability periods without replays is provided in Table 1. Note that all values differ significantly from the Betti numbers exhibited by the coactivity complexes with replays (see Results).

## ACKNOWLEDGMENTS

This document was prepared as an account of work sponsored by the United States Government. While this document is believed to contain correct information, neither the United States Government nor any agency thereof, nor the Regents of the University of California, nor any of their employees, makes any warranty, express or implied, or assumes any legal responsibility for the accuracy, completeness, or usefulness of any information, apparatus, product, or process disclosed, or represents that its use would not infringe privately owned rights. Reference herein to any specific commercial product, process, or service by its trade name, trademark, manufacturer, or otherwise, does not necessarily constitute or imply its endorsement, recommendation, or favoring by the United States Government or any agency thereof, or the Regents of the University of California. The views and opinions of authors expressed herein do not necessarily state or reflect those of the United States Government or any agency thereof or the Regents of the University of California.

This manuscript has been authored by an author at Lawrence Berkeley National Laboratory under Contract No. DE-AC02-05CH11231 with the U.S. Department of Energy. The U.S. Government retains, and the publisher, by accepting the article for publication, acknowledges, that the U.S. Government retains a non-exclusive, paid-up, irrevocable, world-wide license to publish or reproduce the published form of this manuscript, or allow others to do so, for U.S. Government purposes.

## AUTHOR CONTRIBUTIONS

Andrey Babichev: Formal analysis; Investigation; Software; Validation; Visualization. Dmitriy Morozov: Methodology; Software. Yuri Dabaghian: Conceptualization; Data curation; Formal analysis; Funding acquisition; Investigation; Methodology; Project administration; Resources; Software; Supervision; Validation; Visualization; Writing – original draft; Writing – review & editing.

## FUNDING INFORMATION

Yuri Dabaghian, National Science Foundation (http://dx.doi.org/10.13039/100000001), Award ID: 1422438. Dmitriy Morozov, Department of Energy, Office of Science (http://dx.doi.org/10.13039/100006132), Contract ID: DE-AC02-05CH11231.

## TECHNICAL TERMS

Hippocampal place cells: Cells that fire in specific restricted locations in the environmenttheir respective place fields. It is believed that a cognitive map of a given environment emerges from the combined activity of place cells.
Cognitive map: An internal neural representation of the environment, used by the animals (e.g., birds and mammals) to orient in space, to plan spatial navigation strategies, path integrate, and so forth. It is generally accepted that the hippocampus plays a key role in producing and sustaining cognitive maps.
Hippocampal cell assemblies: Transient, functionally interconnected groups of place cells that exhibit frequent coactivity. The organized activity of cells in the hippocampal and cortical assemblies is believed to underlie learning and memory mechanisms.
Replays of place cell sequences: The endogenous activities in the hippocampal network that recapitulate sequential place cell firing during physical exploration of an environment. Spontaneous replays are observed during sleep, quiescent wakeful states, and active navigation. It is believed that replays mark animals exploration and retrieval of the learned spatial information by “cuing” the hippocampal network.

## References

[bib1] Aleksandrov P. (1965). Elementary concepts of topology. New York: Ungar.

[bib2] Alvernhe A., Sargolini F., & Poucet B. (2012). Rats build and update topological representations through exploration. Animal Cognition, 15(3), 359–368. 21915695 10.1007/s10071-011-0460-z

[bib3] Arai M., Brandt V., & Dabaghian Y. (2014). The effects of theta precession on spatial learning and simplicial complex dynamics in a topological model of the hippocampal spatial map. PLoS Computational Biology, 10(6), e1003651. 24945927 10.1371/journal.pcbi.1003651 PMC4063672

[bib4] Atallah B. V., & Scanziani M. (2009). Instantaneous modulation of gamma oscillation frequency by balancing excitation with inhibition. Neuron, 62(4), 566–577. 19477157 10.1016/j.neuron.2009.04.027 PMC2702525

[bib5] Babichev A., Cheng S., & Dabaghian Y. A. (2016). Topological schemas of cognitive maps and spatial learning. Frontiers in Computational Neuroscience, 10, 18. 27014045 10.3389/fncom.2016.00018 PMC4781836

[bib6] Babichev A., & Dabaghian Y. (2017a). Persistent memories in transient networks. In Emergent Complexity from Nonlinearity, in Physics, Engineering and the Life Sciences (pp. 179–188). Cham, Switzerland: Springer.

[bib7] Babichev A., & Dabaghian Y. (2017b). Transient cell assembly networks encode stable spatial memories. Scientific Reports, 7(1), 3959. 28638123 10.1038/s41598-017-03423-3 PMC5479874

[bib8] Babichev A., & Dabaghian Y. A. (2018). Topological schemas of memory spaces. Frontiers in Computational Neuroscience, 12, 27. 29740306 10.3389/fncom.2018.00027 PMC5928258

[bib9] Babichev A., Ji D., Mémoli F., & Dabaghian Y. A. (2016). A topological model of the hippocampal cell assembly network. Frontiers in Computational Neuroscience, 10, 50. 27313527 10.3389/fncom.2016.00050 PMC4889593

[bib10] Babichev A., Morozov D., & Dabaghian Y. (2018). Robust spatial memory maps encoded by networks with transient connections. PLoS Computational Biology, 14(9), e1006433. 30226836 10.1371/journal.pcbi.1006433 PMC6161922

[bib11] Barbieri R., Frank L. M., Nguyen D. P., Quirk M. C., Solo V., Wilson M. A., & Brown E. N. (2004). Dynamic analyses of information encoding in neural ensembles. Neural Computation, 16(2), 277–307. 15006097 10.1162/089976604322742038

[bib12] Bartos M., Vida I., & Jonas P. (2007). Synaptic mechanisms of synchronized gamma oscillations in inhibitory interneuron networks. Nature Reviews Neuroscience, 8(1), 45–56. 17180162 10.1038/nrn2044

[bib13] Basso E., Arai M., & Dabaghian Y. (2016). Gamma synchronization influences map formation time in a topological model of spatial learning. PLoS Computational Biology, 12(9), e1005114. 27636199 10.1371/journal.pcbi.1005114 PMC5026372

[bib14] Best P. J., White A. M., & Minai A. (2001). Spatial processing in the brain: the activity of hippocampal place cells. Annual Review of Neuroscience, 24(1), 459–486. 10.1146/annurev.neuro.24.1.459 11283318

[bib15] Bi G.-Q., & Poo M.-M. (1998). Synaptic modifications in cultured hippocampal neurons: Dependence on spike timing, synaptic strength, and postsynaptic cell type. Journal of Neuroscience, 18(24), 10464–10472. 9852584 10.1523/JNEUROSCI.18-24-10464.1998 PMC6793365

[bib16] Billeh Y. N., Schaub M. T., Anastassiou C. A., Barahona M., & Koch C. (2014). Revealing cell assemblies at multiple levels of granularity. Journal of Neuroscience Methods, 236, 92–106. 25169050 10.1016/j.jneumeth.2014.08.011

[bib17] Burgess N., & O’keefe J. (1996). Cognitive graphs, resistive grids, and the hippocampal representation of space. The Journal of General Physiology, 107(6), 659–662. 8783069 10.1085/jgp.107.6.659 PMC2219393

[bib18] Buzsaki G. (2006). Rhythms of the Brain. Oxford, UK: Oxford University Press.

[bib19] Buzsáki G. (2010). Neural syntax: Cell assemblies, synapsembles, and readers. Neuron, 68(3), 362–385. 21040841 10.1016/j.neuron.2010.09.023 PMC3005627

[bib20] Carlsson G. (2009). Topology and data. Bulletin of the American Mathematical Society, 46(2), 255–308.

[bib21] Carlsson G., & De Silva V. (2010). Zigzag persistence. Foundations of Computational Mathematics, 10(4), 367–405.

[bib22] Carlsson G., De Silva V., & Morozov D. (2009). Zigzag persistent homology and real-valued functions. In Proceedings of the Twenty-Fifth Annual Symposium on Computational Geometry (pp. 247–256).

[bib23] Carr M. F., Jadhav S. P., & Frank L. M. (2011). Hippocampal replay in the awake state: A potential substrate for memory consolidation and retrieval. Nature Neuroscience, 14(2), 147–153. 21270783 10.1038/nn.2732 PMC3215304

[bib24] Chen Z., Gomperts S. N., Yamamoto J., & Wilson M. A. (2014). Neural representation of spatial topology in the rodent hippocampus. Neural Computation, 26(1), 1–39. 24102128 10.1162/NECO_a_00538 PMC3967246

[bib25] Colgin L. L. (2016). Rhythms of the hippocampal network. Nature Reviews Neuroscience, 17(4), 239–249. 26961163 10.1038/nrn.2016.21 PMC4890574

[bib26] Curto C., & Itskov V. (2008). Cell groups reveal structure of stimulus space. PLoS Computational Biology, 4(10), e1000205. 18974826 10.1371/journal.pcbi.1000205 PMC2565599

[bib27] Dabaghian Y. (2016). Maintaining consistency of spatial information in the hippocampal network: A combinatorial geometry model. Neural Computation, 28(6), 1051–1071. 27137840 10.1162/NECO_a_00840 PMC6223651

[bib28] Dabaghian Y., Brandt V. L., & Frank L. M. (2014). Reconceiving the hippocampal map as a topological template. Elife, 3, e03476. 25141375 10.7554/eLife.03476 PMC4161971

[bib29] Dabaghian Y., Mémoli F., Frank L., & Carlsson G. (2012). A topological paradigm for hippocampal spatial map formation using persistent homology. PLoS Computational Biology, 8(8), e1002581. 22912564 10.1371/journal.pcbi.1002581 PMC3415417

[bib30] Dragoi G., & Tonegawa S. (2011). Preplay of future place cell sequences by hippocampal cellular assemblies. Nature, 469(7330), 397–401. 21179088 10.1038/nature09633 PMC3104398

[bib31] Ego-Stengel V., & Wilson M. A. (2010). Disruption of ripple-associated hippocampal activity during rest impairs spatial learning in the rat. Hippocampus, 20(1), 1–10. 19816984 10.1002/hipo.20707 PMC2801761

[bib32] Fenton A. A., Csizmadia G., & Muller R. U. (2000). Conjoint control of hippocampal place cell firing by two visual stimuli: I. The effects of moving the stimuli on firing field positions. The Journal of General Physiology, 116(2), 191–210. 10919866 10.1085/jgp.116.2.191 PMC2229496

[bib33] Foster D. J., & Wilson M. A. (2006). Reverse replay of behavioural sequences in hippocampal place cells during the awake state. Nature, 440(7084), 680–683. 16474382 10.1038/nature04587

[bib34] Fusi S., Asaad W. F., Miller E. K., & Wang X.-J. (2007). A neural circuit model of flexible sensorimotor mapping: Learning and forgetting on multiple timescales. Neuron, 54(2), 319–333. 17442251 10.1016/j.neuron.2007.03.017 PMC2833020

[bib35] Gerrard J. L., Kudrimoti H., McNaughton B. L., & Barnes C. A. (2001). Reactivation of hippocampal ensemble activity patterns in the aging rat. Behavioral Neuroscience, 115(6), 1180–1192. 11770050

[bib36] Girardeau G., Benchenane K., Wiener S. I., Buzsáki G., & Zugaro M. B. (2009). Selective suppression of hippocampal ripples impairs spatial memory. Nature Neuroscience, 12(10), 1222–1223. 19749750 10.1038/nn.2384

[bib37] Girardeau G., & Zugaro M. (2011). Hippocampal ripples and memory consolidation. Current Opinion in Neurobiology, 21(3), 452–459. 21371881 10.1016/j.conb.2011.02.005

[bib38] Goldman-Rakic P. S. (1995). Cellular basis of working memory. Neuron, 14(3), 477–485. 7695894 10.1016/0896-6273(95)90304-6

[bib39] Gothard K. M., Skaggs W. E., & McNaughton B. L. (1996). Dynamics of mismatch correction in the hippocampal ensemble code for space: Interaction between path integration and environmental cues. Journal of Neuroscience, 16(24), 8027–8040. 8987829 10.1523/JNEUROSCI.16-24-08027.1996 PMC6579211

[bib40] Harris K. D., Csicsvari J., Hirase H., Dragoi G., & Buzsáki G. (2003). Organization of cell assemblies in the hippocampus. Nature, 424(6948), 552–556. 12891358 10.1038/nature01834

[bib41] Hasselmo M. E. (2008). Temporally structured replay of neural activity in a model of entorhinal cortex, hippocampus and postsubiculum. European Journal of Neuroscience, 28(7), 1301–1315. 18973557 10.1111/j.1460-9568.2008.06437.x PMC2634752

[bib42] Hatcher A. (2002). Algebraic Topology. Cambridge, UK: Cambridge UP.

[bib43] Hiratani N., & Fukai T. (2014). Interplay between short-and long-term plasticity in cell-assembly formation. PloS One, 9(7), e101535. 25007209 10.1371/journal.pone.0101535 PMC4090127

[bib44] Hoffman K., Babichev A., & Dabaghian Y. (2016). A model of topological mapping of space in bat hippocampus. Hippocampus, 26(10), 1345–1353. 27312850 10.1002/hipo.22610

[bib45] Hopfield J. J. (2010). Neurodynamics of mental exploration. Proceedings of the National Academy of Sciences, 107(4), 1648–1653. 10.1073/pnas.0913991107 PMC2824418 20080534

[bib46] Huxter J. R., Senior T. J., Allen K., & Csicsvari J. (2008). Theta phase–specific codes for two-dimensional position, trajectory and heading in the hippocampus. Nature Neuroscience, 11(5), 587–594. 18425124 10.1038/nn.2106

[bib47] Jadhav S. P., Kemere C., German P. W., & Frank L. M. (2012). Awake hippocampal sharp-wave ripples support spatial memory. Science, 336(6087), 1454–1458. 22555434 10.1126/science.1217230 PMC4441285

[bib48] Jadhav S. P., Rothschild G., Roumis D. K., & Frank L. M. (2016). Coordinated excitation and inhibition of prefrontal ensembles during awake hippocampal sharp-wave ripple events. Neuron, 90(1), 113–127. 26971950 10.1016/j.neuron.2016.02.010 PMC4824654

[bib49] Ji D., & Wilson M. A. (2007). Coordinated memory replay in the visual cortex and hippocampus during sleep. Nature Neuroscience, 10(1), 100–107. 17173043 10.1038/nn1825

[bib50] Johnson A., & Redish A. D. (2007). Neural ensembles in CA3 transiently encode paths forward of the animal at a decision point. Journal of Neuroscience, 27(45), 12176–12189. 17989284 10.1523/JNEUROSCI.3761-07.2007 PMC6673267

[bib51] Jonsson J. (2008). Simplicial Complexes of Graphs (Vol. 3). New York: Springer.

[bib52] Karlsson M. P., & Frank L. M. (2008). Network dynamics underlying the formation of sparse, informative representations in the hippocampus. Journal of Neuroscience, 28(52), 14271–14281. 19109508 10.1523/JNEUROSCI.4261-08.2008 PMC2632980

[bib53] Karlsson M. P., & Frank L. M. (2009). Awake replay of remote experiences in the hippocampus. Nature Neuroscience, 12(7), 913–918. 19525943 10.1038/nn.2344 PMC2750914

[bib54] Knierim J. J., Kudrimoti H. S., & McNaughton B. L. (1998). Interactions between idiothetic cues and external landmarks in the control of place cells and head direction cells. Journal of Neurophysiology, 80(1), 425–446. 9658061 10.1152/jn.1998.80.1.425

[bib55] Koene R. A., & Hasselmo M. E. (2008). Reversed and forward buffering of behavioral spike sequences enables retrospective and prospective retrieval in hippocampal regions CA3 and CA1. Neural Networks, 21(2–3), 276–288. 18242057 10.1016/j.neunet.2007.12.029 PMC2408666

[bib56] Kudrimoti H. S., Barnes C. A., & McNaughton B. L. (1999). Reactivation of hippocampal cell assemblies: Effects of behavioral state, experience, and EEG dynamics. Journal of Neuroscience, 19(10), 4090–4101. 10234037 10.1523/JNEUROSCI.19-10-04090.1999 PMC6782694

[bib57] Kuhl B. A., Shah A. T., DuBrow S., & Wagner A. D. (2010). Resistance to forgetting associated with hippocampus-mediated reactivation during new learning. Nature Neuroscience, 13(4), 501–506. 20190745 10.1038/nn.2498 PMC2847013

[bib58] Leutgeb J. K., Leutgeb S., Treves A., Meyer R., Barnes C. A., McNaughton B. L., … Moser E. I. (2005). Progressive transformation of hippocampal neuronal representations in “morphed” environments. Neuron, 48(2), 345–358. 16242413 10.1016/j.neuron.2005.09.007

[bib59] Louie K., & Wilson M. A. (2001). Temporally structured replay of awake hippocampal ensemble activity during rapid eye movement sleep. Neuron, 29(1), 145–156. 11182087 10.1016/s0896-6273(01)00186-6

[bib60] Lum P. Y., Singh G., Lehman A., Ishkanov T., Vejdemo-Johansson M., Alagappan M., … Carlsson G. (2013). Extracting insights from the shape of complex data using topology. Scientific Reports, 3, 01236. 10.1038/srep01236 PMC3566620 23393618

[bib61] Mizuseki K., Sirota A., Pastalkova E., & Buzsáki G. (2009). Theta oscillations provide temporal windows for local circuit computation in the entorhinal-hippocampal loop. Neuron, 64(2), 267–280. 19874793 10.1016/j.neuron.2009.08.037 PMC2771122

[bib62] Moser E. I., Kropff E., & Moser M.-B. (2008). Place cells, grid cells, and the brain’s spatial representation system. Annual Review of Neuroscience, 31, 69–89. 10.1146/annurev.neuro.31.061307.090723 18284371

[bib63] Muller R. U., Stead M., & Pach J. (1996). The hippocampus as a cognitive graph. The Journal of General Physiology, 107(6), 663–694. 8783070 10.1085/jgp.107.6.663 PMC2219396

[bib64] Murre J. M., Chessa A. G., & Meeter M. (2013). A mathematical model of forgetting and amnesia. Frontiers in Psychology, 4, 76. 23450438 10.3389/fpsyg.2013.00076 PMC3584298

[bib65] Nádasdy Z., Hirase H., Czurkó A., Csicsvari J., & Buzsáki G. (1999). Replay and time compression of recurring spike sequences in the hippocampus. Journal of Neuroscience, 19(21), 9497–9507. 10531452 10.1523/JNEUROSCI.19-21-09497.1999 PMC6782894

[bib66] O’Keefe J., & Dostrovsky J. (n.d.). The hippocampus as a spatial map: Preliminary evidence from unit activity in the freely-moving rat. Brain Research, 171–175. 10.1016/0006-8993(71)90358-1 5124915

[bib67] O’Neill J., Senior T., & Csicsvari J. (2006). Place-selective firing of CA1 pyramidal cells during sharp wave/ripple network patterns in exploratory behavior. Neuron, 49(1), 143–155. 16387646 10.1016/j.neuron.2005.10.037

[bib68] O’Neill J., Senior T. J., Allen K., Huxter J. R., & Csicsvari J. (2008). Reactivation of experience-dependent cell assembly patterns in the hippocampus. Nature Neuroscience, 11(2), 209–215. 18193040 10.1038/nn2037

[bib69] Papale A. E., Zielinski M. C., Frank L. M., Jadhav S. P., & Redish A. D. (2016). Interplay between hippocampal sharp-wave-ripple events and vicarious trial and error behaviors in decision making. Neuron, 92(5), 975–982. 27866796 10.1016/j.neuron.2016.10.028 PMC5145752

[bib70] Pastalkova E., Itskov V., Amarasingham A., & Buzsáki G. (2008). Internally generated cell assembly sequences in the rat hippocampus. Science, 321(5894), 1322–1327. 18772431 10.1126/science.1159775 PMC2570043

[bib71] Petri G., Expert P., Turkheimer F., Carhart-Harris R., Nutt D., Hellyer P. J., & Vaccarino F. (2014). Homological scaffolds of brain functional networks. Journal of The Royal Society Interface, 11(101), 20140873. 10.1098/rsif.2014.0873 PMC4223908 25401177

[bib72] Roux L., Hu B., Eichler R., Stark E., & Buzsáki G. (2017). Sharp wave ripples during learning stabilize the hippocampal spatial map. Nature Neuroscience, 20(6), 845–853. 28394323 10.1038/nn.4543 PMC5446786

[bib73] Russo E., & Durstewitz D. (2017). Cell assemblies at multiple time scales with arbitrary lag constellations. Elife, 6, e19428. 28074777 10.7554/eLife.19428 PMC5226654

[bib74] Sadowski J. H., Jones M. W., & Mellor J. R. (2011). Ripples make waves: Binding structured activity and plasticity in hippocampal networks. Neural Plasticity, 2011, 960389. 21961073 10.1155/2011/960389 PMC3180853

[bib75] Sadowski J. H., Jones M. W., & Mellor J. R. (2016). Sharp-wave ripples orchestrate the induction of synaptic plasticity during reactivation of place cell firing patterns in the hippocampus. Cell Reports, 14(8), 1916–1929. 26904941 10.1016/j.celrep.2016.01.061 PMC4785795

[bib76] Singer A. C., Carr M. F., Karlsson M. P., & Frank L. M. (2013). Hippocampal SWR activity predicts correct decisions during the initial learning of an alternation task. Neuron, 77(6), 1163–1173. 23522050 10.1016/j.neuron.2013.01.027 PMC3751175

[bib77] Singh G., Memoli F., Ishkhanov T., Sapiro G., Carlsson G., & Ringach D. L. (2008). Topological analysis of population activity in visual cortex. Journal of Vision, 8(8), 11–18. 10.1167/8.8.11 PMC2924880 18831634

[bib78] Touretzky D. S., Weisman W. E., Fuhs M. C., Skaggs W. E., Fenton A. A., & Muller R. U. (2005). Deforming the hippocampal map. Hippocampus, 15(1), 41–55. 15390166 10.1002/hipo.20029

[bib79] Wang Y., Markram H., Goodman P. H., Berger T. K., Ma J., & Goldman-Rakic P. S. (2006). Heterogeneity in the pyramidal network of the medial prefrontal cortex. Nature Neuroscience, 9(4), 534–542. 16547512 10.1038/nn1670

[bib80] Wills T. J., Lever C., Cacucci F., Burgess N., & O’keefe J. (2005). Attractor dynamics in the hippocampal representation of the local environment. Science, 308(5723), 873–876. 15879220 10.1126/science.1108905 PMC2680068

[bib81] Wilson M. A., & McNaughton B. L. (1994). Reactivation of hippocampal ensemble memories during sleep. Science, 265(5172), 676–679. 8036517 10.1126/science.8036517

[bib82] Yoganarasimha D., Yu X., & Knierim J. J. (2006). Head direction cell representations maintain internal coherence during conflicting proximal and distal cue rotations: Comparison with hippocampal place cells. Journal of Neuroscience, 26(2), 622–631. 16407560 10.1523/JNEUROSCI.3885-05.2006 PMC1388189

[bib83] Zeithamova D., Schlichting M. L., & Preston A. R. (2012). The hippocampus and inferential reasoning: Building memories to navigate future decisions. Frontiers in Human Neuroscience, 6, 70. 22470333 10.3389/fnhum.2012.00070 PMC3312239

[bib84] Zenke F., & Gerstner W. (2017). Hebbian plasticity requires compensatory processes on multiple timescales. Philosophical Transactions of the Royal Society B: Biological Sciences, 372(1715). 10.1098/rstb.2016.0259 PMC5247595 28093557

