# Supplementary material for: Replays of spatial memories suppress topological fluctuations in cognitive map
Source: Netw Neurosci. 2019 Jul 1;3(3):707–24. doi: 10.1162/netn_a_00076 (PMC6663216; doi:10.1162/netn_a_00076)
Supplement: Supplementary file 1 [file netn-03-707-s001.pdf]

Babichev, A., Morozov, D., & Dabaghian, Y. (2019). Supporting information for "Replays of spatial memories suppress topological fluctuations in cognitive map." *Network Neuroscience*, *3*(3), 707-724. https://doi.org/10.1162/netn_a_00076

Replays of spatial memories suppress topological fluctuations in cognitive map: Supplementary Materials

Andrey Babichev[1], Dmitriy Morozov[2] and Yuri Dabaghian[3*]

SUPPLEMENTARY FIGURES

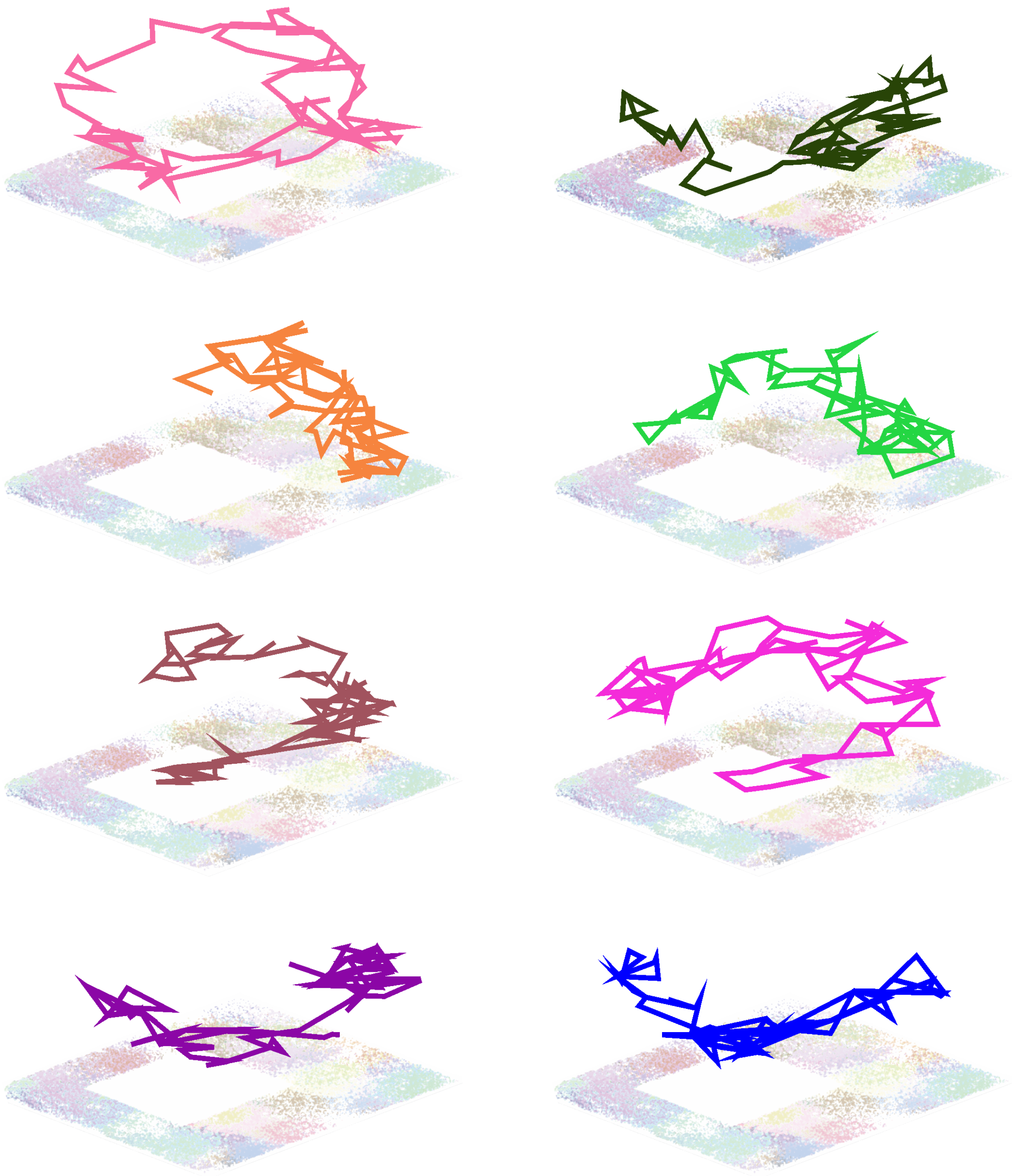

Figure 1: **Replayed trajectories.** Eight simulated trajectories of length $l_s = 100$, which corresponds to about 25 secs of of physical time, extending across different segments of the environment. The positions of the replayed trajectories over the environment are not related to the location of the rat at the moment when the replays occurred.

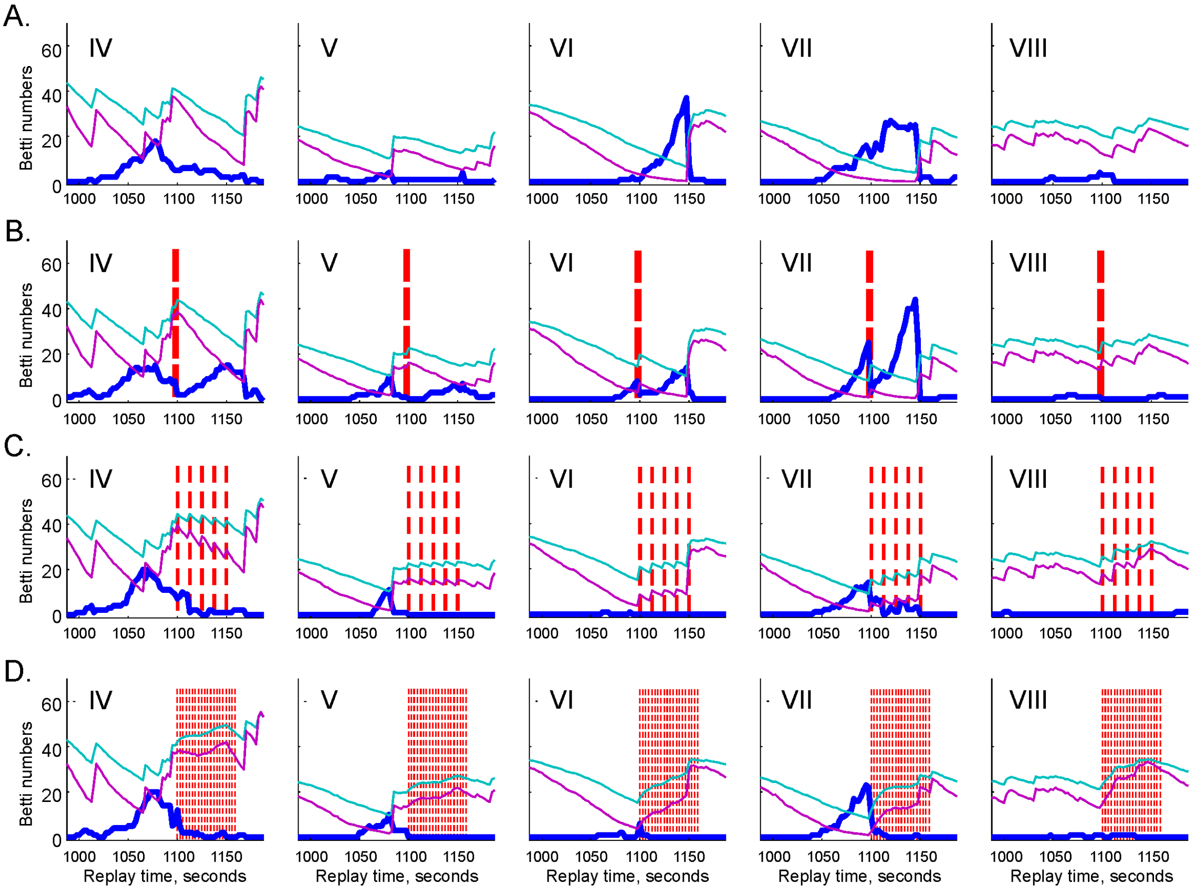

Figure 2: **Local topological fluctuations.** **A**. The first Betti number, b_1 (thick blue line) computed for the sectors IV-VIII of the environment, enumerated according to Fig. 2D. The changes in numbers of cliques and in Betti numbers over the segments I, II and III are insignificant and are not shown. The blue and the magenta lines represent the numbers of pair and triple connections, $N_2(\mathcal{F}_\tau)$ and $N_3(\mathcal{F}_\tau)$, scaled down by the factors 10^{-3} and 10^{-4} respectively, to fit into the panels. Prior to the instability period, the numbers of $N_3(\mathcal{F}_\tau)$ and $N_2(\mathcal{F}_\tau)$ decrease, indicating that the coactivity complex $\mathcal{F}_\tau$ thins out, which also produces an increasing number of spurious topological loops. In contrast, the increase of the local Betti numbers b_1 stops when the connections start to accumulate, i.e., when the decay of the coactivity simplexes is counterbalanced by their regular reactivation due to the rat's uniform sampling of the environment. **B**. In the case of instantaneous massive replay (memory flash, time marked by the vertical red dashed line), the increase of b_1 is briefly halted over all segments of the environment, but then it restarts at the same rate. **C**. For a more frequent replays ($N_r = 5$, times marked by the five red vertical dashed lines), spurious loops are suppressed in all sectors, most effectively over the sectors VI and VII. **D**. In the case of a more regular replay ($N_r = 20$ vertical dashed lines), spurious loops are effectively terminated in all sectors over the entire instability period.

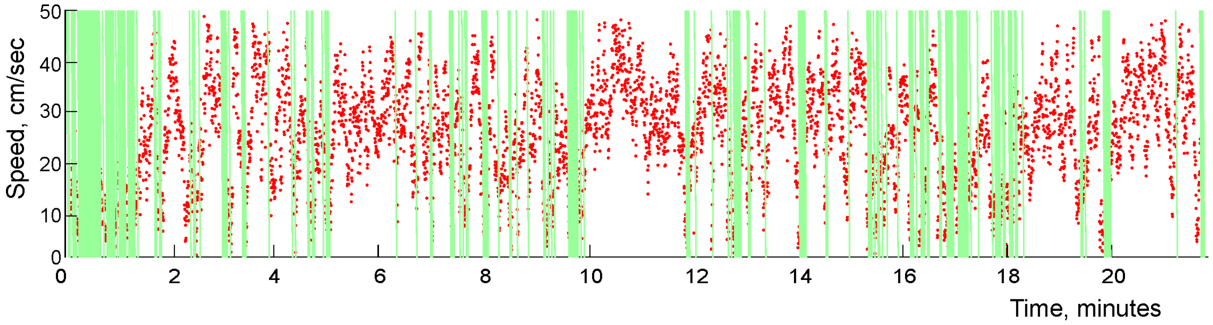

Figure 3: **Speed modulation of the replays.** The red dots mark the mean values of the animal's speed during the coactivity windows—the potential replay times. The green lines mark the slow motion periods, $v < 15$ cm/sec, occurring during 14% of time—the replay windows.

SUPPLEMENTARY MOVIE CAPTIONS

Suppl. Movie 1. Single replay. First two panels show the dynamics of the spatial histograms of the two-vertex and the three-vertex simplexes (i.e., centers of the pairwise and the triple overlaps between place fields) present in $\mathcal{F}_{\tau}$. The right panel shows the trajectory (green line) in the square environment split into eight segments (see Fig. 2C,D). The numbers in each segment S_i represent the pair local Betti numbers $(b_0(S_i), b_1(S_i))$ and the pair of the global Betti number $(b_0(\mathcal{E}), b_1(\mathcal{E}))$ are shown in the center. Around $t = 24$ secs, a large number of replayed sequences is injected (see Fig. 3B). The topological fluctuations are instantaneously suppressed but then they immediately restart and reach back to high values.

Suppl. Movie 2. Five replays suppress the topological fluctuations better (Fig. 3C).

Suppl. Movie 3. Twenty replays (about one replay in every 9 seconds) nearly extinguish the topological fluctuations during the instability period (Fig. 3D).

Suppl. Movie 4. Additional speed-modulation revives topological fluctuations (Fig. 4C,D).

Suppl. Movie 5. Speed-modulated, randomized replays restore the correct topological shape of the map (Fig. 4F).
