# Supplementary figures and images for: Replays of spatial memories suppress topological fluctuations in cognitive map
Source: Netw Neurosci. 2019 Jul 1;3(3):707–24. doi: 10.1162/netn_a_00076 (PMC6663216; doi:10.1162/netn_a_00076)

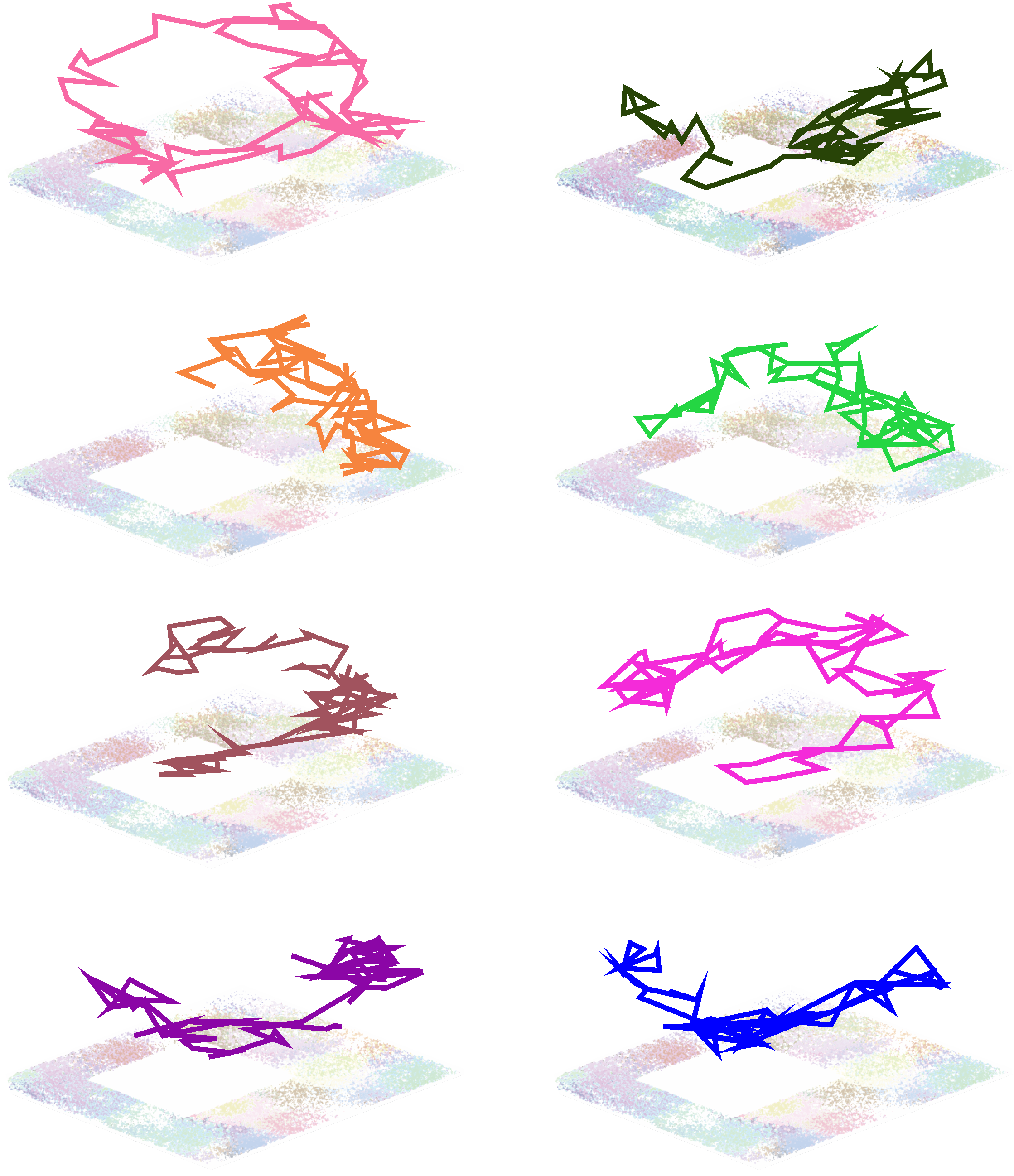

Supplement: Supplementary file 2 [file netn-03-707-s001.tiff]

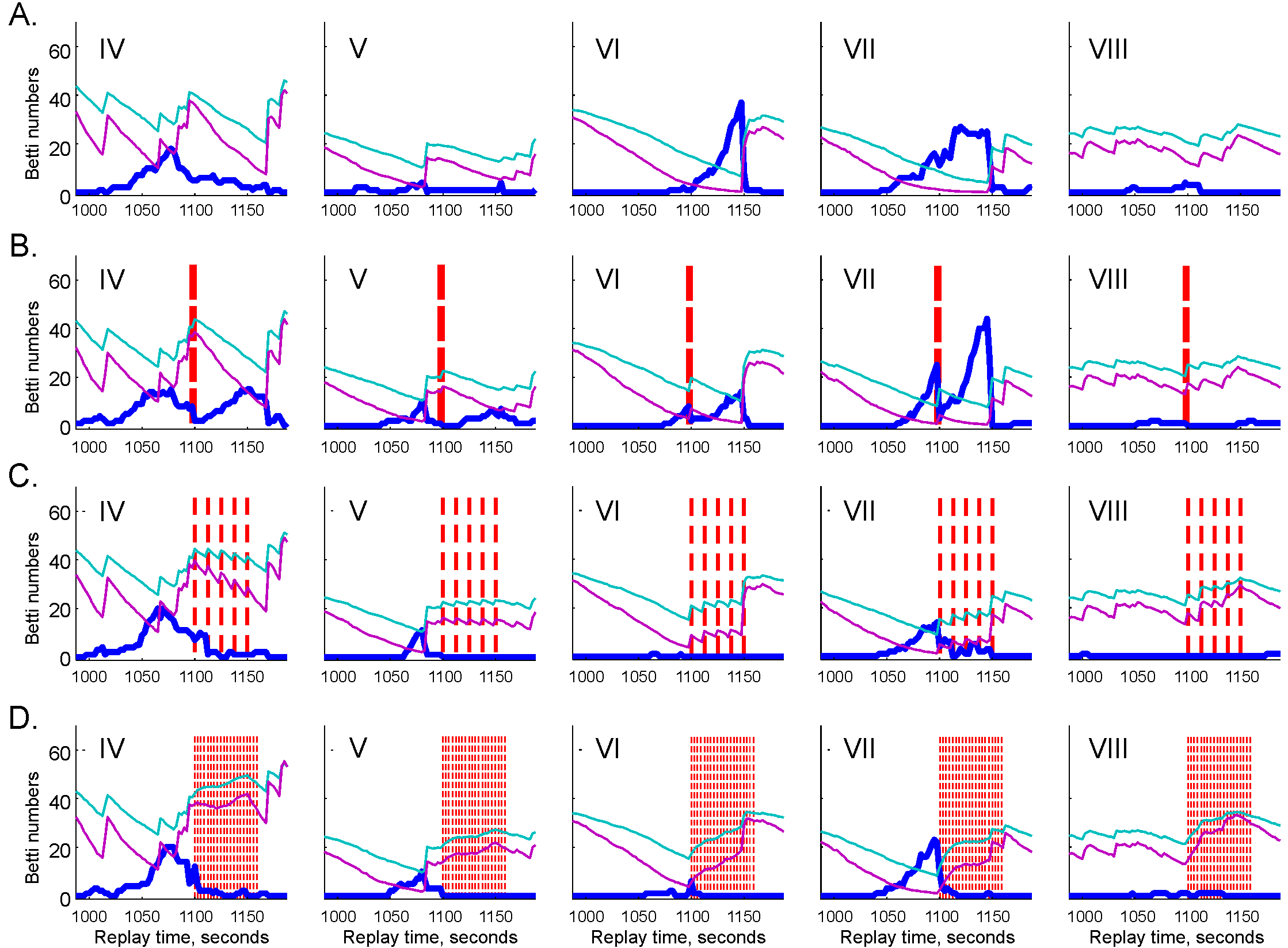

Supplement: Supplementary file 3 [file netn-03-707-s002.tiff]

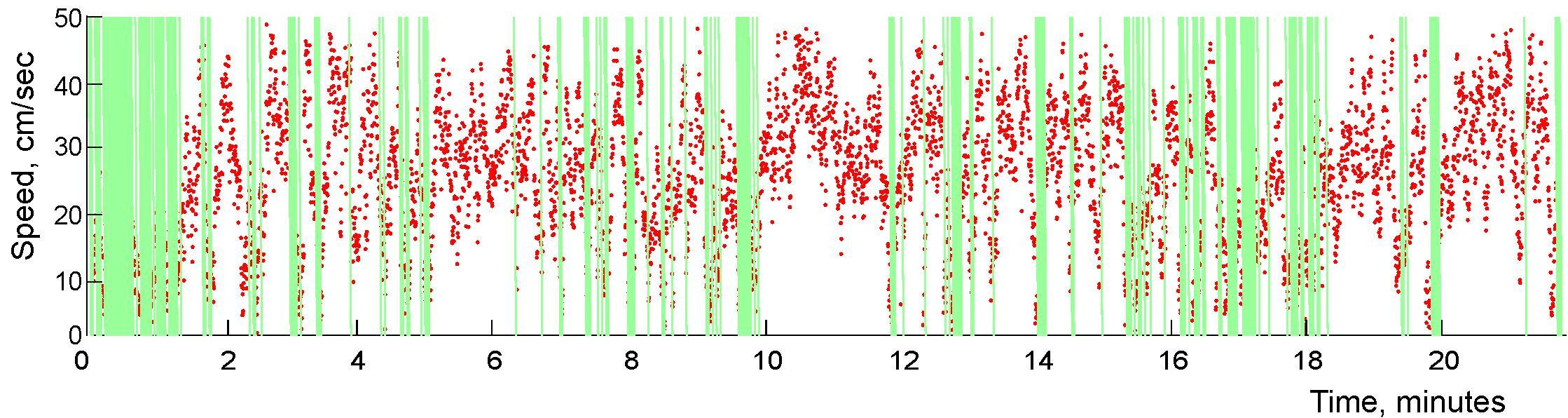

Supplement: Supplementary file 4 [file netn-03-707-s003.tiff]
